# Human Papillomavirus Typing in HIV-Positive Women

**DOI:** 10.1155/S1064744901000163

**Published:** 2001

**Authors:** Meera Hameed, Helen Fernandes, Joan Skurnick, Dorothy Moore, Patricia Kloser, Debra Heller

**Affiliations:** 1 Department of Pathology and Laboratory Medicine New Jersey Medical School 150 Bergen Street-UH-E141 Newark NJ 07103 USA; 2 Department of Preventive Medicine and Community Health New Jersey Medical School Newark NJ USA; 3 Department of Medicine New Jersey Medical School Newark NJ USA

## Abstract

*Objective:* Human papillomavirus (HPV) is the major cause of cervical carcinoma and cervical intraepithelial neoplasia worldwide. Certain HPV types have a strong association with and probably a causative role in the pathogenesis of premalignant cervical lesions. Epidemiologic studies in women infected by the human immunodeficiency virus (HIV) have shown an increased incidence of squamous intraepithelial lesions (SILs), whichwere predominantly high-grade. Six to 30 per cent of women diagnosed with atypical squamous cells of undetermined significance (ASCUS) on a Papanicolaou (Pap) smear harbor SIL in normal screening populations. This study was undertaken to determine the presence of low- and high-risk HPV types in women infected by HIV and to correlate the results to those of the Pap smear.

*Study design:* HPV DNA typing (low- and high-risk) by Digene™ (Digene Corporation, Gathesburg, MD) hybrid capture methodology was performed on cervical swabs from 209 HIV-positive women. The results of HPV typing were correlated with those of the Pap smear in a retrospective analysis.

*Results:* One hundred and one women (48%) tested positive for HPV subtypes by DNA typing by the hybrid capture method. Of these, 64 patients (63%) had Pap smears whichwere read as being normal, having benign cellular changes, or having ASCUS (favor reactive process). Of these, 19 patients tested positive for both high-risk and low-risk subtypes, 32 patients tested positive only for high-risk subtypes, and 13 patients tested positive only for low-risk subtypes.

*Conclusion:* HPV subtyping identifies a significant group of HIV-positive women who are at risk for developing cervical intraepithelial neoplasia, although they may not show significant abnormalities on their Pap smears.


Infect Dis Obstet Gynecol 2001;9:89–93

### Human papillomavirus typing in HIV-positive women

**Meera Hameed[1], Helen Fernandes[1], Joan Skurnick[2], Dorothy Moore[1], Patricia Kloser[3] and Debra Heller[1]**

*[1]Department of Pathology and Laboratory Medicine, [2]Department of Preventive Medicine and Community Health and [3]Department of Medicine, New Jersey Medical School, Newark, NJ*

***Objective:*** Human papillomavirus (HPV) is the major cause of cervical carcinoma and cervical intraepithelial neoplasia worldwide. Certain HPV types have a strong association with and probably a causative role in the pathogenesis of premalignant cervical lesions. Epidemiologic studies in women infected by the human immunodeficiency virus (HIV) have shown an increased incidence of squamous intraepithelial lesions (SILs), which were predominantly high-grade. Six to 30 per cent of women diagnosed with atypical squamous cells of undetermined significance (ASCUS) on a Papanicolaou (Pap) smear harbor SIL in normal screening populations. This study was undertaken to determine the presence of low- and high-risk HPV types in women infected by HIV and to correlate the results to those of the Pap smear.

***Study design:*** HPV DNA typing (low- and high-risk) by Digene™ (Digene Corporation, Gathesburg, MD) hybrid capture methodology was performed on cervical swabs from 209 HIV-positive women. The results of HPV typing were correlated with those of the Pap smear in a retrospective analysis.

***Results:*** One hundred and one women (48%) tested positive for HPV subtypes by DNA typing by the hybrid capture method. Of these, 64 patients (63%) had Pap smears which were read as being normal, having benign cellular changes, or having ASCUS (favor reactive process). Of these, 19 patients tested positive for both high-risk and low-risk subtypes, 32 patients tested positive only for high-risk subtypes, and 13 patients tested positive only for low-risk subtypes.

***Conclusion:*** HPV subtyping identifies a significant group of HIV-positive women who are at risk for developing cervical intraepithelial neoplasia, although they may not show significant abnormalities on their Pap smears.

Key words: HUMAN PAPILLOMAVIRUS (HPV), HUMAN IMMUNODEFICIENCY VIRUS (HIV), ATYPICAL SQUAMOUS CELLS OF UNCERTAIN SIGNIFICANCE (ASCUS), BENIGN CELLULAR CHANGES (BCC), SQUAMOUS INTRAEPITHELIAL LESION (SIL)

Human papillomaviruses (HPV) are small DNA viruses that infect epithelial cells and induce a variety of squamous proliferative lesions[1]. They display tremendous diversity with more than 100 different subtypes described in the literature[2]. It is well known that the majority of cervical carcinomas (more than 90%) harbor human papillomaviruses[3] and a causative role has been implicated[4]. The viral subtypes are classified into low-risk and high-risk based on their ability to induce virus-associated tumors. The high-risk subtypes (HPV 16, 18, 31, 33, 35, 45, 51, 52 and 56) are associated with cervical intraepithelial neoplasia that may progress to carcinoma[5]. In the current Bethesda system for cytology screening (Pap smear), the most common abnormal result is

Correspondence to: Debra Heller, MD, Department of Pathology, New Jersey Medical School, 150 Bergen Street-UH-E141, Newark, NJ 07103. E-mail: hellerds@umdnj.edu one of uncertainty, termed 'atypical squamous cells of uncertain significance' (ASCUS). In the United States about 2 million ASCUS Pap smear results are reported every year[6]. Of these, any proportion between 6 and 30% can harbor squamous intraepithelial lesions (SILs)[7].

Women infected with human immunodeficiency virus (HIV) show an increased incidence of SILs and the majority of these are high-grade SILs as reported by epidemiologic studies[8]. This study was undertaken to correlate the presence of low- and high-risk subtype HPV with Pap smear results in HIV-positive women.

### MATERIALS AND METHODS

Approval from the institutional review board (IRB) was obtained prior to initiation of this study.

Two hundred and nine HIV-positive women who were being monitored by HIV viral load and for whom HPV typing had been performed by the Digene™ (Digene Corporation, Gathesburg, MD) hybrid capture method as a routine clinical test were selected from a database at the Molecular Diagnostics Laboratory at the New Jersey Medical School. The corresponding Pap smear results were obtained from the Division of Cytology at the same institution and the results were correlated with the findings of HPV typing. All normal, benign cellular changes, and ASCUS results were reconfirmed by one of us (DH).

### HPV typing by Digene hybrid capture

The Digene hybrid capture HPV DNA assay is a hybridization antibody capture assay using chemiluminescence to qualitatively detect the presence of 14 HPV subtypes (6, 11, 16, 18, 31, 33, 35, 42, 43, 44, 45, 51, 52 and 56). The assay is designed to distinguish between two HPV DNA groups, low-risk (6, 11, 42, 43 and 44) and high-intermediate risk (16, 18, 31, 33, 35, 45, 51, 52 and 56), in cervical specimens (cervical swabs and fresh cervical biopsies). Briefly, specimens containing the target DNA hybridize with a specific HPV RNA probe cocktail. The resultant RNA:DNA hybrids are captured onto the surface of a tube coated with antibodies specific for RNA:DNA hybrids. Immobilized hybrids are then reacted with alkaline phosphatase-conjugated antibodies specific for the RNA:DNA hybrids, and then detected with a chemiluminescent substrate. As the substrate is cleaved by the bound alkaline phosphatase, light is emitted which is measured as relative light units (RLUs) on a luminometer. The intensity of the light emitted denotes the presence or absence of target DNA in the specimen. Computations were performed using StatXact® software (Cytel, Cambridge, MA). Ninety-five per cent confidence intervals based on the binomial distribution were computed for estimates of proportions with positive and negative results.

### RESULTS

One hundred and one patients (48%) tested positive for HPV (either low- or high-risk) subtypes. Of these, the Pap smear results were read as normal, or revealed benign cellular changes or ASCUS (favor reactive process), in 64 patients (63%). Of the 64 patients, 19 (30%) were positive for both high-risk and low-risk subtypes, an additional 32 (50%) tested positive for high-risk HPV subtypes, and the remaining 13 patients (20%) tested positive for low-risk subtypes.

Twenty-four of the 209 patients (11%) had low-grade SIL on Pap smear. Among the low-grade SIL patients, 12 tested positive for high-risk subtypes only, two for low-risk subtypes only, and nine for both subtypes. One patient with low-grade SIL tested negative for HPV subtyping. Twelve patients (0.06%) had Pap smears diagnosed as ASCUS (favor dysplasia). Of these, seven showed positivity for both subtypes, (one for high-risk only) and four tested negative for HPV subtyping. Only three patients showed high-grade SIL, of which two were positive for both subtypes and one for low-risk subtypes only. Of three patients diagnosed on Pap smear with dysplasia (grade cannot be determined), two had only high-risk subtypes and one tested positive for low-risk subtypes only. Results are summarized in Table 1.

Of the 101 HPV-positive patients, 64 (63%) had a Pap smear showing ASCUS (favor reactive process), benign cellular changes, or a normal result. Of these, 45 (45%) had benign cellular changes or normal findings (95% confidence interval (CI) 35–55%) and 19 were read as ASCUS

**Table 1** Correlation between human papillomavirus (HPV) test results and Pap smear findings

| *HPV subtypes* | *Normal/BCC* | *ASCUS (reactive)* | *ASCUS (dysplasia)* | *LGSIL* | *HGSIL* | *Dysplasia/ ?grade* | *Total* |
|---|---|---|---|---|---|---|---|
| Low-risk only | 9 | 4 | 0 | 2 | 1 | 0 | 16 |
| High-risk only | 25 | 7 | 1 | 12 | 0 | 2 | 47 |
| Both high-risk and low-risk subtypes | 11 | 8 | 7 | 9 | 2 | 1 | 38 |
| Total HPV-positive | 45 | 19 | 8 | 23 | 3 | 3 | 101 |
| Both subtypes negative | 97 | 6 | 4 | 1 | 0 | 0 | 108 |

ASCUS, atypical cells of undetermined significance; BCC, benign cellular changes; HGSIL, high-grade squamous intraepithelial lesion; LGSIL, low-grade squamous intraepithelial lesion

(reactive) (95% CI 12–28%). Of the 64 ASCUS (reactive) and/or normal/ benign cellular changes patients, 51 (80%) had high-risk HPV subtypes (95% CI 68–89%).Only 37 HPV-positive patients had abnormal Pap smears, denoting a sensitivity of only 37% (95% CI 27–47%) for Pap smear alone to detect the HPV-positive individuals.

### DISCUSSION

In the past 40 years there has been a dramatic decline in the incidence of cervical carcinoma in the United States due to the widespread use of cytologic screening, which can detect premalignant lesions (SILs) that can be treated successfully. However, there is considerable interlaboratory variability in detection of SILs and up to 20% of SILs can go undetected by routine screening procedures[9–12]. Additionally 5–10% of women with the diagnosis of ASCUS harbor SILs and more than one-third of screening populations who harbor high-grade intraepithelial lesions are identified from ASCUS results[6]. The central role of HPV as a causative phenomenon in cervical intraepithelial neoplasia has been well established by experimental and epidemiologica data[13–15]. The recognition of certain HPV types such as 16, 18, 31, 33, 35, 45, 51, 52 and 56 as underlying etiologic agents for cervical carcinoma has raised the expectation that HPV DNA typing may be of value in identifying women at risk of developing premalignant lesions of the cervix. HPV is detected by PCR analysis in cervical cancers in about 90% of cases[16,17] and the prevalence is strongly correlated to the grade of SIL[18]. It has also been shown that the presence of HPV DNA as determined by dot blot hybridization correlated with the detection of high-grade SIL within 3 years[19].

Women infected with HIV are five times more likely to develop cervical carcinoma than uninfected women[20]. Some studies have suggested that HIV infection increases the strength of association between HPV and SIL and that the viral pathogenic effect is probably mediated through HIV-mediated immune suppression[21,22]. The number of sex partners and CD4 cell count can affect the association between HPV and HIV[23,24]. It has also been shown that the incidence of false negative findings following a single Pap smear in the presence of histologically confirmed SIL or invasive carcinoma could be as high as 50% in HIV-positive women[25].

In this study we found that there is high prevalence of HPV in HIV-positive women (47%), a finding similar to many reports in the literature[26–30]. Sixty-three percent of patients who showed normal or minimally abnormal Pap smear results tested positive for HPV in our study. A similar finding is reported by Uberti-Foppa and colleagues[27]. In their study, 63% of cytologically negative HIV-positive women tested positive for HPV high-risk subtypes by the hybrid capture method. This also supports the view that HPV infection is more persistent in women who are at risk for developing cervical neoplasia than in others[31]. A noteworthy finding in our study is the high prevalence of HPV among patients with normal or benign cellular changes (45 of 101 patients). The majority of these patients also harbored high-risk subtypes (36 of 45) which are similar to the data of Uberti-Foppa and colleagues[27]. The high percentage of HPV

positivity with negative cytology in our study reduces the sensitivity of the Pap smear to 37% for detecting patients at high risk for developing cervical intraepithelial neoplasia in this population of HIV-infected women. Of the 101 HPV-positive patients we tested, 19 showed ASCUS (favor reactive process) by Pap smear. Fifteen of the 19 patients harbored high-risk HPV subtypes. In a large cohort study by Manos and co-workers, HPV DNA testing was recommended for women with ASCUS Pap smear results to help identify those who may have underlying high-grade SIL[6]. In our study, 80% of HPV-positive patients with normal, benign cellular changes, or ASCUS (favor reactive process) Pap smears tested positive for high-risk HPV subtypes. These findings suggest that routine screening of cervical cytology should include HPV subtype identification in HIV-infected women especially in areas where careful cytologic screening over time may not be feasible due to issues such as noncompliance in the screening population. Our results indicate that a Pap smear alone may not exclude HPV infection. Furthermore, a significant number of patients who are at risk of cervical intraepithelial neoplasia can be identified by HPV DNA subtyping in addition to the Pap smear. This approach would also ensure appropriate follow-up and treatment of cervical SIL in a timely fashion in HIV-positive women where rapid progression of disease is a strong possibility.

### REFERENCES

1. Alani MR, Munger K. Human papilloma viruses and associated malignancies. *J Clin Oncol* 1998; 16:330–7
2. Raymond K, Ervin A, Vladimir V. Human papillomavirus infection and cervical carcinoma. *Clin Obstet Gynecol* 2000;43:363–80
3. DeVilliers EM. Heterogeneity of the human papilloma virus group. *J Virol* 1989;63:4898–903
4. Farthing A, Masterson P, Mason WP, Vousden KH. Human papilloma virus detection by hybrid capture and its possible clinical use. *J Clin Pathol* 1994;47:649–52
5. Lorincz A, Temple GF, Kurman RJ, *et al*. Oncogenic association of specific human papillomavirus types with cervical neoplasia. *J Natl Cancer Inst* 1987;79:671–7
6. Manos MM, Kinney WK, Hurley LB, *et al*. Identifying women with cervical neoplasia. Using human papillomavirus DNA testing for equivocal Papanicolaou results. *JAMA* 1999;281:1605–10
7. Crum CP, Genest DR, Krane JF, *et al*. Subclassifying atypical squamous cells in thin-prep cervical cytology correlates with detection of high-risk human papillomavirus DNA. *Am J Clin Pathol* 1999;112:384–90
8. Wright TC, Sun XW. Anogenital papillomavirus infection and neoplasia in immunodeficient women. *Obstet Gynecol* 1996;23:861–93
9. Cox JT, Lorincz AT, Schiffman MH, *et al*. Human papillomavirus testing by hybrid capture appears to be useful in triaging women with a cytological diagnosis of atypical squamous cells of undetermined significance. *Am J Obstet Gynecol* 1995; 172:946–54
10. Reid R, Greenberg MD, Lorincz A, *et al*. Should cervical cytological testing be augmented by cervicography or human papillomavirus deoxyribonucleic acid detection? *Am J Obstet Gynecol* 1991;164:1461–71
11. Wright TC, Sun XW, Koulos J. Comparison of management algorithms for the evaluation of women with low grade cytologic abnormalities. *Obstet Gynecol* 1995;85:202–10
12. Davey DD, Naryshkin S, Nielsen ML, Kline TS. Atypical squamous cells of undetermined significance; interlaboratory comparison and quality assurance of monitors. *Diagn Cytopathol* 1994; 11:390–6
13. zur Hausen H, de Villiers EM, Gissman L. Papillomavirus infections and human genital cancer. *Gynecol Oncol* 1981;12(Suppl 1):124–8
14. International Agency Research on Cancer. *The Epidemiology of Human Papillomavirus and Cervical Cancer*. International Agency Research on Cancer scientific monograph 119. Lyon, France: IARC, 1992
15. Schiffman MH. Recent progress in defining the epidemiology of human papillomavirus infection and cervical neoplasia. *J Natl Cancer Inst* 1992; 84:394–8
16. Fujinaga Y, Shimada M, Okazawa K, *et al*. Simultaneous detection and typing of genital human papillomavirus DNA using polymerase chain reaction. *J Gen Virol* 1991;72:1039–44

17. Kiyabu MT, Shibata D, Arnheim N, *et al.* Detection of human papillomavirus in formalin fixed invasive squamous carcinomas using the polymerase chain reaction. *Am J Surg Pathol* 1989;13:221–4
18. Cuzick J, Terry G, Hollingworth T, Anderson M. Human papilloma virus type 16 in cervical smears as predictor of high grade cervical intraepithelial neoplasia. *Lancet* 1992;339:959–60
19. Koutsky LA, Holmes KK, Critchlow CW, *et al.* A cohort study of the risk of cervical intraepithelial neoplasia grade 2 or 3 in relation to papillomavirus infection. *N Engl J Med* 1992;327:1272–8
20. Mandelblatt JS, Fahs M, Garibaldi K, *et al.* Association between HIV infection and cervical neoplasia: implications of clinical care of women at risk for both conditions. *AIDS* 1992;6:173–8
21. Laga M, Icenogle JI, Marsella R, *et al.* Genital papillomavirus infection and cervical dysplasia – opportunistic complication of HIV infection. *Int J Cancer* 1992;50:45–8
22. Schrager LK, Friedland GHD, Maude Schreiber K, *et al.* Cervical and vaginal squamous cell abnormalities in women infected with human immunodeficiency virus. *J AIDS* 1989;2:570–3
23. Piper MA, Severin ST, Wiktor AZ, *et al.* Association of human papilloma virus with HIV and CD4 cell count in women with high or low numbers of sex partners. *Sex Transmiss Inf* 1999;75:253–7
24. Vernon SD, Unger ER, Piper MA, *et al.* HIV and human papillomavirus as independent risk factors for cervical neoplasia in women with high or low numbers of sex partners. *Sex Transmiss Inf* 1999;75:258–60
25. Syrjanen S, Saastamoinen J, Chang F, *et al.* Colposcopy, punch biopsy, *in situ* DNA hybridization and the polymerase chain reaction in searching for genital human papillomavirus (HPV) infections in women with normal Pap smears. *J Med Virol* 1990;31:259–66
26. Rezza G, Giuliani M, Branca M, *et al.,* and the DIANAIDS Collaborative Study Group. Determinants of squamous intraepithelial lesions (SIL) on Pap smear: the role of HPV infection and of HIV-1 induced immunosuppression. *Eur J Epidemiol* 1997;13:937–43
27. Uberti-Foppa C, Origoni M, Maillard M, *et al.* Evaluation of the detection of human papillomavirus genotypes in cervical specimens by hybrid capture as screening for precancerous lesions in HIV positive women. *J Med Virol* 1998;56:133–7
28. Ho GYF, Burk RD, Fleming I, Klein RS. Risk of genital human papillomavirus infection in women with human immunodeficiency virus induced immunosuppression. *Int J Cancer* 1994;56:788–92
29. Tweddel G, Heller P, Cunnane M, *et al.* The correlation between HIV seropositivity, cervical dysplasia and HPV sub types 6/11, 16/18, 31/33/35. *Gynecol Oncol* 1994; 52:161–4
30. Vernon SD, Holmes KK, Reeves WC. Human papillomavirus infection and associated disease in persons infected with human immunodeficiency virus. *Clin Infect Dis* 1995;21:121–4
31. Hildesheim A, Schiffman MH, Gravitt PE, *et al.* Persistence of type specific human papillomavirus infection among cytologically normal women. *J Infect Dis* 1994;169:235–40

Received 09/05/00; Accepted 01/19/01
